# Exploring the Application of Contrast‐Enhanced Ultrasound in ACR BI‐RADS 4A Lesions (≤ 2 cm): A Prospective Multicenter Study in China

**DOI:** 10.1155/tbj/6622181

**Published:** 2026-03-13

**Authors:** Xiaoyun Xiao, Wei Zhang, Xiaomao Luo, Yulan Peng, Yanling Zheng, Qi Zhou, Qiao Hu, Ying Zhu, Heng Zhang, Yinghua Li, Qing Zhou, Wen He, Baoming Luo

**Affiliations:** ^1^ Department of Ultrasound, Sun Yat-sen Memorial Hospital, Sun Yat-sen University, Guangzhou, China, sysu.edu.cn; ^2^ Department of Ultrasound, Beijing Tiantan Hospital, Capital Medical University, Beijing, China, ccmu.edu.cn; ^3^ Department of Ultrasound, Yunnan Cancer Hospital, The Third Affiliated Hospital of Kunming Medical University, Kunming, China, kmmc.cn; ^4^ Department of Ultrasound, West China Hospital, Sichuan University, Chengdu, China, scu.edu.cn; ^5^ Department of Ultrasound, The First Affiliated Hospital of Sun Yat-sen University, Guangzhou, China, sysu.edu.cn; ^6^ Department of Ultrasound, The Second Affiliated Hospital of Xi’an Jiaotong University, Xi an, China, xjtu.edu.cn; ^7^ Department of Ultrasound, The People’s Hospital of Guangxi Zhuang Autonomous Region, Nanning, China, gxhospital.com; ^8^ Department of Ultrasound, Tianjin Medical University Cancer Institute & Hospital, Tianjin, China, tijmu.edu.cn; ^9^ Department of Ultrasound, Zhuhai People’s Hospital, Zhuhai Hospital Affiliated to Jinan University, Zhuhai, China, zhhospital.cn; ^10^ Department of Ultrasound, Yan’an Hospital of Kunming City, Yan’an Hospital Affiliated to Kunming Medical University, Kunming, China; ^11^ Department of Ultrasound, Renmin Hospital of Wuhan University, Wuhan, China, rmhospital.com

**Keywords:** ACR BI-RADS, breast, contrast-enhanced ultrasound, neoplasm, ultrasonography

## Abstract

**Purpose:**

To assess the added diagnostic value of contrast‐enhanced ultrasound (CEUS) in differentiating small breast lesions initially categorized as ACR BI‐RADS 4A.

**Materials and Methods:**

A total of 1595 patients with small breast lesions (≤ 20 mm) from 37 tertiary hospitals were enrolled from August 2021 to August 2022. B‐mode ultrasound, color Doppler, and CEUS were performed to evaluate the lesions. The integration of CEUS led to the reclassification of BI‐RADS 4A lesions into three pathways: upgrade to 4B, downgrade to 3, or no change in category. The diagnostic performance of CEUS was evaluated, and the contrast modes which could be used to correctly downgrade or upgrade BI‐RADS 4A lesions were explored.

**Results:**

A total of 1340 lesions were finally included in the analysis of CEUS performance. The average age of all included participants was 43 ± 8 (range from 20 to 84). The average diameter of the lesions was 12.4 ± 3.8 mm (range from 5 mm to 20 mm). The diagnostic performances of CEUS + BI‐RADS were 98.3% for sensitivity, 81.7% for specificity, 34.1% for positive predictive value, 99.8% for negative predictive value, and 83.1% for overall accuracy, respectively. The sensitivity was slightly downgraded (98.3% vs. 100%, *p* = 0.1555), while the specificity and accuracy were greatly elevated (*p* < 0.05). The receiver operating characteristic curve (ROC) value was calculated using a binary classification threshold, with BI‐RADS 3 as low‐risk and BI‐RADS 4A as high‐risk. The area under ROC was 0.896 (95% CI: 0.878, 0.911) for BI‐RADS + CEUS. The decision curve analysis showed that using CEUS + BI‐RADS to guide biopsy decisions provided net benefit compared to the default strategy of biopsying all lesions.

**Conclusion:**

The addition of CEUS allows for the correct downgrading of most benign BI‐RADS 4A lesions, thereby avoiding unnecessary biopsies. An upgrade from BI‐RADS 4A to 4B on CEUS warrants immediate biopsy to ensure prompt diagnosis and treatment.

**Trial Registration:** Chinese Clinical Trial Registry: ChiCTR2100050719

**Key Messages**
• Patients with small ACR BI‐RADS 4A lesions (≤ 20 mm) would benefit from contrast‐enhanced ultrasound according to this multicenter study. It reduces unnecessary biopsies and allows for the early detection of malignant lesions.

## 1. Introduction

According to Breast Imaging Reporting and Data System released by American College of Radiology (ACR BI‐RADS), biopsy is recommended for women with breast lesions categorized as ACR BI‐RADS 4A, even though the likelihood of malignancy is only 2%–10% [1]. In a populous nation like China, with its 1.5 billion inhabitants, routine screening programs are identifying a growing number of women diagnosed with BI‐RADS 4A lesions. Whether all such cases necessitate biopsy remains a subject of ongoing debate. Consequently, how to further refine the risk stratification of BI‐RADS 4A lesions has consistently been a key concern for clinicians.

Sonographers have been actively working on addressing this issue and have made notable progress. Ultrasound artificial intelligence was reported to reveal more subtle differences of ultrasound morphological and texture characteristics between benign and malignant BI‐RADS 4A lesions [2, 3]. Shear wave elastography could help discriminate low‐suspicion lesions from benign lesions to decrease false‐positive findings and avoid missing cancer diagnoses [4]. A nomogram incorporating clinical factors and imaging characteristics was reported to be applicable for downgrading low‐risk lesions in BI‐RADS 4A [5, 6].

As is widely recognized, angiogenesis is a critical process for the growth of malignant tumors. The use of contrast media allows for a superior depiction of this microcirculation, facilitating the visualization of the vessel network induced by vascular endothelial growth factor. Hence, contrast‐enhanced ultrasound (CEUS) could elevate the specificity of breast ultrasound [7], even in tiny lesions [8]. CEUS has been applied in the differentiation of breast lesions for over 30 years [7, 9, 10]. Nevertheless, CEUS is still an undergoing active research field which is yet to be recommended for clinical use according to the EFSUMB guidelines 2017 [11].

Larger lesions tend to exhibit more typical 2D characteristics on B‐mode ultrasound, thereby facilitating a more confident diagnosis. For relatively small breast lesions (≤ 20 mm), especially those categorized as BI‐RADS 4A, 2D characteristics were not specific enough for differentiation. Several studies have demonstrated the efficiency of CEUS in this group of patients [12, 13]. For example, Lin et al. raised a principal component regression‐based CEUS evaluation system [13]. It should be noted, however, that these data were analyzed in separate centers with relatively small sample sizes. This prompts the question of whether the findings are replicable and reliable in a larger, more diverse cohort. Accordingly, we undertook this multicenter study with the primary aim of assessing the diagnostic validity of CEUS for relatively small (≤ 20 mm) BI‐RADS 4A breast lesions. The objectives of the study were: (1) to figure out whether CEUS could be beneficial for risk stratification of BI‐RADS 4A lesions and (2) to summarize ultrasound characteristics for BI‐RADS 4A lesions.

## 2. Materials and Methods

The prospective multicenter study was registered in the Chinese Clinical Trial Registry (ChiCTR). And it was conducted in accordance with the Declaration of Helsinki (as revised in 2013) and approved by the Medical Ethics Committee of our hospital (No. 2021‐KY‐046) which was the leader unit of this research. All the other hospitals got the approvals from their Medical Ethics Committees. Written consent was obtained from each patient enrolled in the study. The entire study strictly adhered to the STARD guidelines. Initially, over 56 hospitals participated in the multicenter study. After standard imaging‐acquisition training, 37 hospitals with eligible cases were finally engaged in the study from August 2021 to August 2022. The inclusion criteria were: (a) BI‐RADS 4A breast lesion detected in B‐mode ultrasound with a maximal diameter ≤ 20 mm; (b) CEUS was performed before biopsy; and (c) pathology was obtained. The exclusion criteria were: (a) pregnant and parturient women; (b) non‐mass‐like lesions; and (c) lesions located in a breast quadrant that had undergone prior surgery.

The diagnostic criterion for BI‐RADS 4A was based on the literature [14]. A typical benign breast mass (homogeneous, oval, parallel, circumscribed margin, Adler Grade 0 or I) accompanied with one of the following suspicious characteristics could be categorized as BI‐RADS 4A. The suspicious characteristics included: (a) nonparallel, (b) irregular shape, (c) microlobulation, (d) Adler Grade II or III, (e) with a breast cancer family history or detected over 40, and (f) significant enlargement over a short period. All cases were collected by sonographers with at least 5 years of experience in breast ultrasound and 2 years of experience in breast CEUS.

For standardization, all ultrasound machines used in the multicenter study must be equipped with real‐time gray‐scale contrast‐enhanced software and were adjusted to the best settings for breast CEUS. The ultrasound machines used in the multicenter study were ACUSON Sequoia (Siemens Healthineers, Germany), Resona9/Resona7 (Mindray, China), Aplio i900 (Canon, Japan), and LOGIQ E9/Voluson E20 (GE, USA). The contrast agent used in the study was Sonovue (Bracco Imaging B.V., Geneva, Switzerland); 4.8 mL of the contrast medium was injected via the antecubital vein followed by a 10‐mL saline flush.

All lesions were scanned thoroughly. The typical images and dynamic clips included: (a) B‐mode images in two perpendicular planes; (b) dynamic clips based on the B‐mode images mentioned above; (c) color Doppler images of the lesion; (d) CEUS clips of the lesion, and (e) other typical images essential for diagnosis (optional). All static images and dynamic clips were recorded for review.

All patients in this study underwent core needle biopsy or vacuum‐assisted excision within 1 week following CEUS, with pathology serving as the gold standard. No adverse events were reported during or after CEUS and biopsy.

### 2.1. Image Reading

The expert panel consisted of six experts from six different centers. All of them had at least 20 years of experience in breast ultrasound and over 10 years of experience in breast CEUS. Each lesion was judged by two experts. The experts were all blinded to the pathological results of the lesions.

First, all the 1595 lesions were assessed. For every lesion categorized as BI‐RADS 4A, the reviewing expert was required to document the rationale, detailing the specific sonographic features that contributed to this classification. The factors included clinical information (age, personal history, and family history), B‐mode, and color Doppler characteristics (based on Adler’s grades and the distribution of the vessels) of the lesion. Only the lesions categorized as BI‐RADS 4A by both experts were included for final analysis of the performance of breast CEUS.

All the experts made an initial census on judging a contrast‐mode [15–17]. A training set of 30 cases was provided to the experts to calibrate their interpretation of CEUS findings. For each BI‐RADS 4A lesion, two experts were asked to depict the contrast‐enhanced pattern of the lesion. The contrast‐enhanced pattern of the lesion was analyzed from the following 10 indexes, including: (a) enhanced time (compared with breast tissue); (b) enhanced intensity (compared with breast tissue); (c) enhanced direction; (d) homogeneity; (e) margin after enhancement; (f) shape after enhancement; (g) scope after enhancement; (h) ring‐like enhancement; (i) crab‐claw‐like enhancement, and (j) perfusion defect.

The experts were then asked to assess whether integrating CEUS findings would lead to a reclassification of the BI‐RADS category for each lesion. If yes, the lesions were either downgraded to BI‐RADS 3 or upgraded to BI‐RADS 4B. And if not, the lesions remained as BI‐RADS 4A. The contrast patterns used to downgrade a lesion were: (a) hypo‐ and comparatively later enhancement without clear borderline; (b) iso‐ and synchronous enhancement without clear borderline; and (c) hyper‐ and early enhancement with clear borderline and regular shape, and the enhanced scope is equal to or smaller than the 2D one. The contrast mode used to upgrade a lesion was hyperenhancement with larger scope, with or without irregular shape or crab‐claw‐like enhancement.

As per ACR BI‐RADS criteria, lesions rated BI‐RADS 4 or greater should undergo biopsy for pathological evaluation. The criteria for expert interpretation and agreement were explicitly detailed. “Consensus” was defined as both experts assigned the same BI‐RADS classification for one lesion. “Management” was whether the redefined BI‐RADS would affect the next‐step decision‐making, tissue diagnosis, or continued surveillance (Table 1).

**TABLE 1:** Decision agreement between experts.

Expert 1	Expert 2	Consensus	Management
BI‐RADS 3	BI‐RADS 3	Y	Y
BI‐RADS 4A	BI‐RADS 4A	Y	Y
BI‐RADS 4B	BI‐RADS 4B	Y	Y
BI‐RADS 4A	BI‐RADS 4B	N	Y
BI‐RADS 3	BI‐RADS 4A	N	N

*Note:* After experts evaluated the contrast enhancement patterns of the lesions, the lesions were reclassified. When two experts assigned the same BI‐RADS classification, it indicated consensus. “Management” referred to tissue diagnosis or continued surveillance according to ACR BI‐RADS. “Y” meant yes, while “N” meant no.

The lesions recategorized as BI‐RADS 3 were considered as “low‐risk,” while the lesions recategorized as BI‐RADS 4B or remained as BI‐RADS 4A were considered as “high‐risk.” A lesion was only classified as BI‐RADS 3 if both experts independently assessed it as category 3. In all other scenarios, the lesions were classified into the “high‐risk” group.

We performed decision curve analysis (DCA) to evaluate the clinical utility of our model. The *x*‐axis represents the threshold probability for recommending biopsy. At a given threshold probability pt, a lesion would be biopsied if its predicted malignancy probability exceeded pt, otherwise it would be recommended for short‐term follow‐up. We examined threshold probabilities from 2% to 30%, as thresholds below 2% correspond to the BI‐RADS 3 category (where follow‐up is standard).

SPSS (version 23.0) and MedCalc (version 20.0.4) were used for statistical analysis. DCA was carried out within the R environment for statistical computing (version 4.5.0; R Core Team, 2023). Inter‐rater reliability was assessed by Kappa statistics. Chi‐square test was used to evaluate the diagnostic efficiency of CEUS in different age groups. *p* < 0.05 was considered statistically significant.

## 3. Results

**FIGURE 1:**
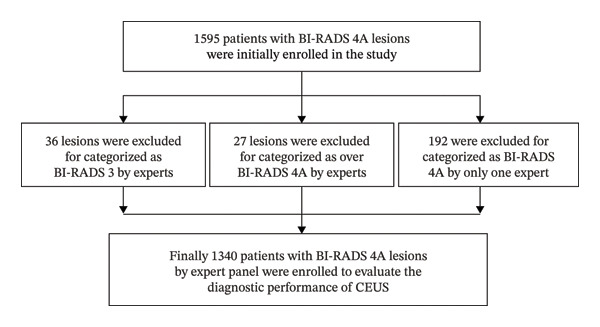
Flowchart of breast lesion enrollment.

From August 2021 to August 2022, a total of 1595 patients with 1595 solid lesions were enrolled in the study at the beginning. After B‐mode images being evaluated by the expert panel, 1340 lesions of 1340 patients were finally included in the analysis of breast CEUS performance. There were 1222 benign lesions and 118 malignant lesions (Figure 1, Table 2). The included cases were over the threshold of the required data calculated as a noninferiority trial (1232 cases). The likelihood of malignancy of the enrolled cases was 8.9%, which was within the range of malignancy likelihood of BI‐RADS 4A (2%–10%). The average age of the patients included was 43 ± 8 (range from 20 to 84). The average diameter of the lesions was 12.4 ± 3.8 mm (range from 5 to 20 mm).

**TABLE 2:** Pathology of breast lesions.

Benign	1222	Malignant	118
Fibroadenoma	599	Invasive ductal carcinoma	80
Fibroadenosis	328	Ductal carcinoma in situ	24
Intraductal papilloma	170	Intraductal papillary carcinoma	5
Sclerosing adenosis	74	Invasive lobular carcinoma	3
Chronic inflammation	19	Malignant phyllodes tumor	2
Benign phyllodes tumor	17	Invasive papillary carcinoma	2
Complicated cyst	13	Mucinous carcinoma	1
Pseudo‐hemangiomatous	1	Metastasis	1
Stromal hyperplasia			
Myofibroblastoma	1		

Among the lesions categorized as BI‐RADS 4A, margin (1011/1340, 75.4%), shape (933/1340, 69.6%), age (527/1340, 39.3%), vascularity (355/1340, 26.5%), duct changes (250/1340, 18.7%), and nonparallel orientation (178/1340, 13.3%) emerged as the six main factors contributing to the decision‐making process. Solid lesions with regular shape and noncircumscribed margin (microlobulated/indistinct) or solid lesions with irregular shape and circumscribed margin, combined with one or two of the following features might be regarded as BI‐RADS 4A. The features included duct changes, nonparallel orientation, Adler II/III, age over 40, and personal or family history of breast cancer.

**FIGURE 2:**
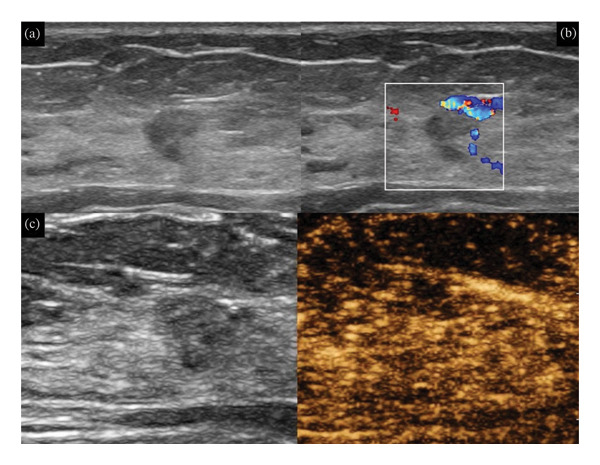
A 28‐year‐old lady with a BI‐RADS 4A lesion in the right breast. The lesion was detected in routine screening without any associated symptom. The maximal diameter of the lesion was 9 mm. In combination with contrast‐enhanced ultrasound, the lesion was downgraded to BI‐RADS 3. Ultrasound‐guided vacuum‐assisted excision was performed. The pathology was adenosis. (a) B‐mode ultrasound showed a hypoechoic lesion with nonparallel orientation and (b) color Doppler detected vessels in rim, and (c) CEUS showed iso‐ and synchronous enhancement with surrounding breast tissue.

**FIGURE 3:**
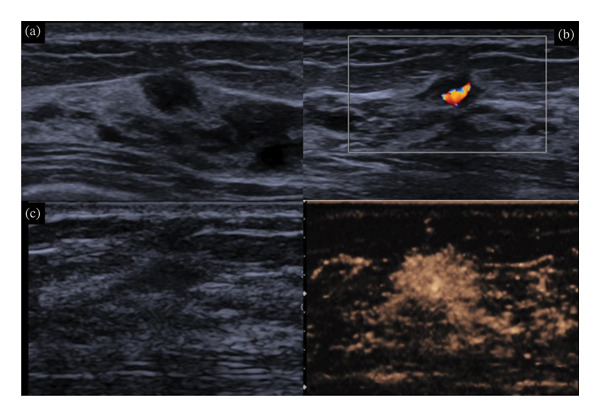
A 42‐year‐old lady with a BI‐RADS 4A lesion in the right breast. The maximal diameter of the lesion was only 6 mm. No discomfort was complained. In combination with contrast‐enhanced ultrasound, the lesion was upgraded to BI‐RADS 4B. Ultrasound‐guided core‐needle biopsy was performed. The pathology was intraductal carcinoma. (a) B‐mode ultrasound showed a hypoechoic lesion with microlobulation margin and (b) color Doppler detected vessels in the lesion, and (c) CEUS showed hyperenhancement with irregular shape and larger scope.

Of the 1340 lesions initially categorized as BI‐RADS 4A, 1000 (74.6%) were downgraded to BI‐RADS 3 following integration of CEUS findings (Figure 2). Among them, 2 malignant lesions were missed (0.2%). The pathology was ductal carcinoma in situ with microinvasion. A total of 998 unnecessary biopsies (74.5% of all the lesions) were avoided; 96 lesions were upgraded to BI‐RADS 4B (Figure 3). There were 17 benign lesions and 79 malignant lesions. Over 66.9% (79/118) malignant lesions were further confirmed by CEUS. Seventeen overdiagnosed benign lesions (1.4% of benign lesions) included 6 cases of intraductal papilloma, 6 cases of sclerosing adenosis, and 5 cases of fibroadenoma. Thirty‐six malignant lesions were still judged as BI‐RADS 4A in combination with CEUS by at least one expert. A total of 244 lesions were with different BI‐RADS categories (not in agreement), and 77 of them were with different decisions (Table 1). There were 2 malignant lesions (DCIS) and 75 benign lesions. Interobserver agreement using Kappa statistics claimed substantial agreement (*κ* = 0.728) between two groups of experts.

According to our established judgment principle, 77 lesions with different management were all classified as “high‐risk.” Taken low risk and high risk as cut‐off point, the diagnostic performances of CEUS + BI‐RADS were 98.3% for sensitivity, 81.7% for specificity, 34.1% for positive predictive value, 99.8% for negative predictive value, and 83.1% for overall accuracy. The sensitivity was slightly downgraded (98.3% vs. 100%, *p* = 0.1555), while the specificity and the accuracy were greatly elevated (*p* < 0.05). The area under receiver operating characteristic curve (ROC) was 0.896 (95% CI: 0.878, 0.911) for BI‐RADS + CEUS.

**FIGURE 4:**
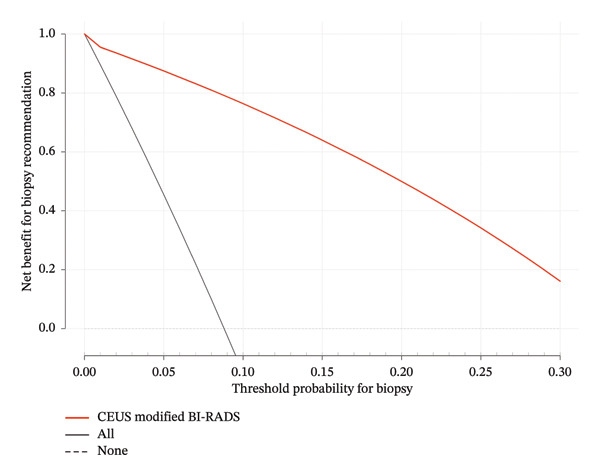
Decision curve analysis for the contrast‐enhanced ultrasound model in guiding biopsy of BI‐RADS 4A lesions. The decision curve analysis evaluates the clinical utility of the contrast‐enhanced ultrasound (CEUS) prediction model. The *x*‐axis represents the threshold probability for recommending biopsy—the minimum predicted malignancy probability at which a clinician would opt for biopsy over short‐term follow‐up. The *y*‐axis represents the standardized net benefit, quantifying the net true positives per 100 patients after accounting for the harm of unnecessary biopsies weighted by the threshold probability.

The DCA showed that using CEUS + BI‐RADS to guide biopsy decisions provided net benefit compared to the default strategies of biopsying all lesions or biopsying none (Figure 4).

Furthermore, the patients were divided into two subgroups according to age (age < 40 and age ≥ 40). The diagnostic performances of CEUS + BI‐RADS in patients aged < 40 showed 100% for sensitivity, 77.3% for specificity, and 78.3% for overall accuracy. While in patients aged ≥ 40, the sensitivity, specificity, and overall accuracy were 97.9%, 85.2%, and 86.6%, respectively. We found no evidence of difference of sensitivities between two groups (*χ* ^2^ = 0.065, *p* = 0.7983). The specificity and overall accuracy differed statistically between two groups (*χ* ^2^ = 11.747, *p* < 0.05 for specificity; *χ* ^2^ = 14.529, *p* < 0.05 for overall accuracy).

## 4. Discussion

According to the ACR BI‐RADS 2013 guidelines, all lesions categorized as BI‐RADS 4 require tissue diagnosis. However, the likelihood of malignancy for BI‐RADS 4A lesions is only 2%–10%, indicating that the majority of these biopsies may ultimately be unnecessary. This issue is compounded by the fact that the diagnostic criteria for distinguishing the BI‐RADS 4 subcategories are not strictly defined. In this study, several B‐mode characteristics for BI‐RADS 4A definition were summarized. Furthermore, CEUS may serve as a valuable adjunct for further stratifying lesions within the BI‐RADS 4A category. Notably, patients over the age of 40 appeared to derive greater diagnostic benefit from this technique.

In this study, although some diagnostic criteria were raised based on previous research [14], the initial enrollment of BI‐RADS 4A lesions relied more on the experience of sonographers. Among the 1595 initially enrolled lesions, 192 were excluded due to a discrepancy in classification between two independent experts, where one assigned a BI‐RADS 4A category while the other rated the same lesion as either BI‐RADS 3 or 4B (Figure 1). The diagnostic criteria for BI‐RADS 4A remain formally undefined, leading to inherent subjectivity and interobserver variability. To address this, our study aimed to investigate the underlying rationale used by clinicians when assigning the BI‐RADS 4A category to breast lesions. What was striking was that only 298 of the 1340 lesions (22.2%) were consistently assigned to BI‐RADS 4A based on identical imaging criteria, while a marked discrepancy was noted in the remaining cases, with 126 lesions (9.4%) being categorized for entirely different reasons. Hence, the classification of the BI‐RADS 4 subcategory was operator‐dependent. A regular lesion with an indistinct or microlobulated margin, a lesion with irregular shape but circumscribed margin, and an irregular lesion with duct changes are the three main B‐mode features defining BI‐RADS 4A in our study. Age and personal/family history were highly accounted for, especially when determining lesions as BI‐RADS 3 or BI‐RADS 4A with similar B‐mode characteristics. Angular and spiculated margins were seldom depicted in BI‐RADS 4A lesions. Previous research showed that lesions with irregular morphology, unclear margin, and nonparallel orientation tended to be malignant [18]. In our cohort, none of the BI‐RADS 4A lesions exhibited all three characteristics. This finding aligns with the established, relatively low malignancy risk associated with this subcategory.

Lin reported that the diagnostic specificity could be elevated combining B‐mode ultrasound with CEUS (0.600 vs. 0.795) [13]. Yu reported a specificity of 86% of CEUS in BI‐RADS 4A breast masses < 20 mm [12]. Our study showed that in combination with CEUS, a large proportion of BI‐RADS 4A lesions could be correctly downgraded with a specificity of 81.7%. This is in accordance with previous studies. Only 2 lesions were missed, which consisted 0.2% of all the lesions downgraded. Both missed lesions were with a maximal diameter of under 10 mm and the pathology was DCIS. The reason might be the lack of abnormal vessels [19]. A delayed diagnosis of DCIS might not have an impact on survival, which allowed short‐term follow‐up instead of biopsy [20]. The malignancy likelihood (0.2%) of reclassified BI‐RADS 3 in our study was significantly lower than that of BI‐RADS 3 lesions (≤ 2%). According to ACR BI‐RADS, such lesions are appropriate for short‐interval follow‐up or continued surveillance. Furthermore, patients with false‐positive results showed increased anxiety and higher depression when at recall [21]. Therefore, downgrading these lesions in combination with CEUS had clinical application significance. Several contrast patterns used to downgrade BI‐RADS 4A lesions were summarized according to our research. They were (a) hypo‐ and comparatively later enhancement without clear borderline; (b) iso‐ and synchronous enhancement without clear borderline; and (c) hyperenhancement and early enhancement with clear borderline and regular shape, and the enhanced scope is equal to or smaller than the 2D one. It meant a lesion with low‐ or iso‐vascularity comparing with normal breast tissue could be judged as benign with great confidence, which was consistent with Liu’s study [22]. In contrast, the CEUS pattern led to upgrade was hyperenhancement with a larger scope, with or without irregular shape or crab‐claw‐like enhancement. The likelihood of malignancy was as high as over 80% in the rerated BI‐RADS 4B lesions. As a result, immediate biopsy was needed for timely diagnosis and treatment.

A total of 167 lesions retained their initial BI‐RADS 4A classification because the observed contrast patterns did not provide sufficient evidence for reclassification. These patterns included (a) hyperenhancement without a clear borderline with surrounding breast tissue, either earlier or synchronous enhanced; (b) hyperenhancement with a clear borderline which was hard to ascertain whether the scope was larger or not by the naked eye (usually isolated lesions without surrounding breast tissue). The likelihood of malignancy of this group was slightly higher than 10% (24/167). Therefore, we recommend short‐term follow‐up as the preferred management strategy. This is particularly advisable for patients over 40 or with lesions larger than 10 mm. However, an immediate biopsy should be considered for patients who have a personal or family history of breast cancer.

For 133 lesions categorized as BI‐RADS 4A by one expert and BI‐RADS 3 by another, all were downgraded to BI‐RADS 3 in combination with CEUS and proved to be benign by pathology. These results imply that in scenarios of discrepant classification between BI‐RADS 4A and 3 based on conventional ultrasound, CEUS provides critical additional information, aiding in final categorization and increasing confidence in subsequent biopsy decisions. The likelihood of malignancy might also increase with age. Hai et al. reported that age was also influential in predicting the malignancy of BI‐RADS 4A lesions [23], as did Niu et al. [5]. Our study further identified patient age over 40 years as a significant determinant in lesion assessment. As age is already a considered factor in the clinical judgment leading to a BI‐RADS 4A categorization, the incremental value of adding CEUS appears to be particularly pronounced in this subgroup. When applying 40 years as a cutoff, CEUS demonstrated a comparatively higher diagnostic performance for patients above this age threshold.

**FIGURE 5:**
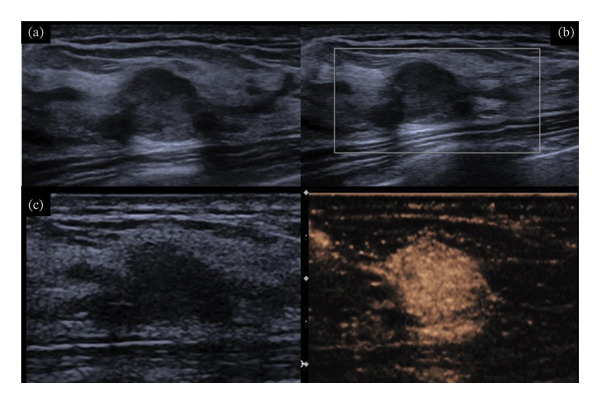
A 47‐year‐old lady with a BI‐RADS 4A lesion in the right breast. The patient reported a possible palpable mass. The maximal diameter of the lesion was 13 mm. In combination with contrast‐enhanced ultrasound, the lesion was downgraded to BI‐RADS 3. Ultrasound‐guided vacuum‐assisted excision was performed. The pathology was intraductal papilloma. (a) B‐mode ultrasound showed a hypoechoic lesion with duct change and posterior enhancement, (b) color Doppler detected no flow signal in or around the lesion, and (c) CEUS showed hyperenhancement with regular shape and similar scope.

Consistent with prior research, this study confirmed that lesion scope, shape, and enhanced intensity remained the principal determinants of CEUS evaluation. [16]. Scope accounted for a great proportion in the final assessment. The judgment of scope enlargement was subjective to some extent when the change was subtle. It might be the clearer outline depicted by contrast agents compared with the blurred margin in 2D images, which confused the judgment (Figure 5). Therefore, for a factor of great significance, objective evaluation by AI might be a better way, which could further elevate the efficacy of CEUS.

This study had several limitations. First, although it is a multicenter study, most cases were collected from Southern China, which might elicit geographical bias. Second, the interpretation of B‐mode images and CEUS was operator‐dependent. The doctors in the expert panel were all with rich experience, which might not represent the average level of all the sonographers in China. Hence, it might limit the generalizability of the study. Standard and feasible training protocols for sonographers and reference image banks for BI‐RADS 4A with CEUS might broaden clinician proficiency. Third, non‐mass‐like breast lesions were not included in the study. Fourth, a very small number of DCIS may be misdiagnosed. Some subtle changes might not be observed by the naked eye. Since artificial intelligence has been proven to be helpful in image processing and feature extracting of CEUS [24], a more stable and standard interpretation might be achieved by combination with artificial intelligence, which might be the next step.

## 5. Conclusions

This study identified specific B‐mode features associated with a higher likelihood of a BI‐RADS 4A classification. When combined with CEUS, most benign lesions within this category could be accurately downgraded, thereby avoiding unnecessary biopsies—a benefit particularly pronounced for patients over 40. Conversely, for the subset of BI‐RADS 4A lesions that were upgraded to BI‐RADS 4B based on CEUS findings, immediate biopsy is recommended to ensure timely diagnosis and management.

**Nomenclature**
ACR BI‐RADS: Breast Imaging Reporting and Data System released by American College of Radiology
CEUS: Contrast‐enhanced ultrasound

## Funding

No funding was received for this manuscript.

## Conflicts of Interest

The authors declare no conflicts of interest.

## Data Availability

The data that support the findings of this study are available upon request from the corresponding author. The data are not publicly available due to privacy or ethical restrictions.
